# Naval casualty management training using human patient simulators

**DOI:** 10.1186/2054-314X-1-9

**Published:** 2015-04-06

**Authors:** Itamar Netzer, Aviram Weiss, David Hoppenstein

**Affiliations:** 3 Medical Department, Haifa Naval Base, Israeli Navy, IDF Medical Corp, Heyl Hayam Square, Haifa, Israel; 4 IDF Medical Corp, Tel Hashomer, Israel; 5 Department of Anesthesia, Critical Care and Pain Management, Meir Medical Center, Kfar Sava, Israel

**Keywords:** Simulation, Casualty care, Naval medicine, Austere environments, Prehospital

## Abstract

**Background:**

Extended-evacuation or austere environments (e.g. naval**,** immature or depleted combat zones) are characterized by the lack of resources to facilitate medical evacuation in the “Golden Hour” from moment of injury. This may require the primary caregiver, often a relatively inexperienced general physician or EMT, to administer extended medical care in the field.

We describe the Shipboard and Underwater Casualty Care and Sedation Simulation (SUCCeSS) program in the Israeli Navy, intended to train caregivers for extended prehospital intensive casualty care using high fidelity life-size simulation mannequins set up onboard corvettes or submarines during maneuvers, in maximally realistic conditions.

Twenty two general physicians and EMTs in 12 teams were enrolled in the program in the years 2011–2013.

Two to three hour long training sessions were headed by senior surgeons and anesthesiologists using flexible scripts enabling the mannequin operators to react to caregivers’ actions and their consequences.

Trainee evaluation was performed by the preceptors using semi-structured forms taking into account both critical treatment decisions and observation on the effects of actions taken. Trainees also completed self-report CRM (Crisis Resource Management) questionnaires before and after the sessions.

**Results:**

Success of the trainees correlated with an evaluation score above 72%. The mean overall CRM score for team leaders post exercise was 74.64%, an improvement of 10% over pre-exercise scores (p < 0.0001).

**Conclusion:**

Caregiver self-perceived competence and self-sufficiency in treating casualties at sea was improved via high fidelity simulation in theatre using realistic naval casualty care situations. We discuss the relative strengths and weaknesses of our training program for the teaching of “NCM”, or Naval Casualty Management, as well as the emergent concepts of the military extended evacuation environment.

**Electronic supplementary material:**

The online version of this article (doi:10.1186/2054-314X-1-9) contains supplementary material, which is available to authorized users.

## Background

This paper presents the SUCCeSS (Shipboard & Underwater Casualty Care & Sedation Simulation) program conducted by the Haifa Naval Base Medical Department with the support of the Maritime Medicine Branch of the Israeli Navy and the Trauma Instruction Section of the Israeli Defense Forces Medical Academy. The program began in 2011, undertaking to train naval trauma teams in Naval Casualty Management (NCM) onboard a ship or submarine at sea using high fidelity medical simulators. Twelve teams have undergone the program over its two years, Each team was led by a military physician (not exceeding General Practitioner by academic training), and included an EMT, a total of 22 personnel trained thus far. Teams were selected from the Israeli Missile Ships and Submarine Flotillas.

The aims of this paper are to delineate the need for extended evacuation training for naval personnel; to demonstrate the feasibility of instituting such a program; and to demonstrate the efficacy of one such program.

The military naval milieu is characterized by relative isolation from immediate logistic support; long distance from Level I trauma centers, and a possible lack of airborne means of evacuation. In the case of combat casualties, this may lead to extended evacuation time and a necessity for continuous critical care. Furthermore, the onboard medical team may be limited in size, with no possibility of shift changes or reinforcements. The caregiver may rely only on limited or depleted resources and supplies at hand, thus demanding a rational utilization of limited resources, and at times requiring some improvisation.

An emergent concept in military trauma care is that of the austere or extended-evacuation environment. Previously, the “Golden Hour” concept of trauma drove caregivers to evacuate casualties to a hospital-based trauma team within 60 minutes of injury by “scoop and run” or “scoop, treat and run” [1]. This has been successfully achieved by the allied forces in Iraq and Afghanistan [2]. The Israeli Defense Forces (IDF) have also successfully achieved this goal in recent conflicts. However, the medical and airborne logistics that must be in place for rapid evacuation to an adequate trauma center are not readily available in immature, winding down, naval or Special Forces theaters of operation. In such environments, the caregiver, be they a medic, EMT, or physician, may find themselves treating the casualty for hours before evacuation to a medical facility. This has slowly led to a paradigm shift in the military medical approach to evacuation. While the concept of the “Golden Hour” is as relevant as ever, steps are being taken by military forces throughout the world to better equip and train medical providers for the eventuality of unavoidable extended care prior to evacuation [3, 4].

Characteristic incidental trauma teams in the Israeli Navy (in contrast with our designated surgical/resuscitation teams) include, in addition to enlisted medics, general practitioners, emergency medical technicians (EMTs) or both. EMTs are by the nature of their training focused on pre-hospital care, having undergone 16 months of EMT training including Tactical Combat Casualty Care (TCCC) and performing regular civilian EMS shifts. Deployed medical officers in the Israeli Defense Force are also trained in combat casualty care. This includes surgical rounds during medical school and internship, A 5 day ATLS course adapted to the military setting, periodic trauma drills in one of the IDF’s Medical Simulation Centers, operational drills practicing trauma care in the military setting and ongoing (albeit brief) hospital trauma training.

However, neither group has extensive training or experience in critical care in an extended-evacuation setting. Both groups are comprised of young, inexperienced caregivers, having little prior experience (no longer than 2 years).

High fidelity life-size simulation mannequins are rapidly gaining acceptance and widespread use in university hospitals and military medical branches alike [5, 6].

Common uses include combat medical training and skills assessment (i.e. in armed forces) and training and competency evaluation elsewhere. Simulation-based training has proven itself highly effective and efficient in improving trauma care skills, both in hospital and prehospital settings, and in the sometimes remote or austere environment of the military setting [7–9].

However, the locale of the classroom or military training facility may neglect to simulate genuine aspects of on-site care, especially as pertains naval medicine - isolation, ambient temperature, heaving of the naval vessel, or cramped quarters. Naval forces employing medical simulation for training of primary caregivers often use labs or remote, littoral facilities in lieu of using naval vessels [10].

## Methods

SimMan II and later, SimMan Essential mannequins, (Laerdal, Stavanger, Norway) were used. Moulage was applied to simulate specific combat injuries. A team of a paramedic-level operator and a senior anesthesiologist/intensive care specialist (“Preceptor”) operated each mannequin. Training sessions were video-recorded and the videos later used for trainee feedback, coupled with the “patient”s” vital signs and procedure scoring. All training sessions took place during naval maneuvers of an Israeli missile corvette or submarine. For a list of resuscitation devices and specifications please refer to Appendix 1.

Prior to the training session at sea, all teams underwent a day-long presail “priming” session in order to become familiarized with the mannequins, equipment, and doctrine. Classes were given on the subjects of sedation and treatment of shipboard medical crises, covering nearly all common scenarios. To promote standardization, Preceptors were briefed in the use of the training facilities (simulators, scripts and evaluation tools). The authors were available on hand in all the sessions, and cross-consultations were made in real time to ensure correct course of the exercises

As an added measure of realism and contrary to the teams’ prior experience, simulation mannequins would “expire” due to incorrect critical treatment decisions resulting in probable human death, i.e. “Dead is Dead”. This was done to motivate the teams and prevent the false reassurance of a “reboot-able patient”.

The teams were trained and assessed in the elements and scenarios listed in Table 1.

**Table 1:** **life support elements addressed in training**

Advanced trauma life support elements	Medical (non trauma) elements	Technical elements	Pharmacological elements
Airway management	Arrhythmias	Loss of electrical power supply	Management of sedation
Tension pneumothorax	Anaphylaxis	Use of adjunct devices – NGT, Foley catheter, intercostal drain	Fluid and blood product resuscitation
Blast injuries	Hypothermia	Ventilator malfunction/disconnection	Toxic gas inhalation (CO, CN)
Electrocution (leading to VF, rhabdomyolysis)	Management of the severe burns patient		
Head Injury	Supportive (i.e. nursing) care		
Smoke inhalation	Prolonged care of the casualty in the absence of immediate evacuation (all sessions lasted 3.5 hours).		
Triage			

Twenty two primary caregivers were trained over the program’s three years. Of these, 15 were physicians and 7 were EMTs.

### Flexible scripts

Scenario descriptions began with initial history and condition of the casualty. Further events and complications were suggested in each scenario based on time elapsed and possible trainee actions. However, we gave the preceptors allowance for divergence from the written scenario. They were encouraged to do this if it was felt that the individual trainee made a questionable treatment decision or if a learning opportunity arose. The mannequin operator would then simulate changes in the mannequin’s vital signs or symptoms based on the preceptor’s suggestions. Additional suggestions for script events were made throughout the training sessions by the authors overseeing the exercise, reflecting treatment decisions of the trainees. The basic scenarios used were:

1. While working on a mast a sailor falls and sustains head injuries. The apparent cause of his fall is electrocution.
2. Following an explosion in one of the sections, a sailor presents with chest trauma and smoke inhalation.

“Additional file 1” contains example scenarios.

In addition to preplanned complications, the teams had to deal with complications arising throughout the treatment due to errors in patient management or incorrect management techniques. Problems such as pulmonary edema from fluid overload in burn patients and misdiagnosis of drug induced anaphylaxis in ventilated patients as airway obstruction, were encountered and would have to be dealt with successfully for the simulated patient to survive.

### Evaluation

Evaluation of medical simulation sessions is a challenging area having a plethora of evaluation devices, at different levels of validation. There exist dozens of clinical skill evaluation tools, most without reported reliability or constant validity. While consensus in the medical education community seems to point to the mini-CEX (Clinical Evaluation Exercise) as a valid and reliable assessment tool [11], its use in medical simulations has scarcely been researched. In addition, the nature of the exercises was formative rather than summative, i.e. the caregivers were being trained rather than being tested. Thus, the authors composed a novel tool relevant to the goals of the SUCCeSS program. Its purpose was to assist the preceptors in recording and assessing the trainees’ actions. In addition, one other team offered peer-based critique in every exercise.

The primary evaluation questionnaire comprised two sections (see Additional file 2 section for the forms used):

I. Critical Treatment Decisions
II. Observation - Effect of actions taken.

The critical treatment decision (CTD) section included nine care decisions generally agreed to be pivotal in resuscitation, and suited to the scenario at hand. Each CTD was marked Yes/No. Failure to identify and perform the CTD elicited a possible deterioration of the casualty, to the point of demise. The CTD section served to record and evaluate the trainee’s decision-making process and his or her understanding of the casualty’s pathophysiology in life-threatening conditions. The focus of the session was employment of a rational algorithm-based approach to the casualty as opposed to trial and error. The CTDS for each session were tailored to the script and scenario being practiced. Preceptors were encouraged to lead the exercise to each of the CTD junctions described in the script and evaluation form.

The observation on effects of actions taken included nine to ten possible interventions performed on the casualty. The possible observations were “Not Undertaken”, “Deleterious”, “Indifferent” or “Beneficial”. This section aimed to evaluate the trainees’ technical skills and medical knowledge, in non-critical treatment actions. For example, a failed intubation attempt on a patient judged in error to be apneic would not be beneficial, and would be valued as deleterious or indifferent, as the case may be.

In both sections the primary subject of the evaluation was the primary caregiver who also led the team.

In addition, teamwork was subjectively evaluated in terms of leadership, cooperation, and inclusion of all team members in the care process. The debriefing section of each exercise emphasized critical treatment decisions and failures, and focused on teamwork and leadership exhibited during the drill.

Team leaders also provided self-evaluation using a validated self-efficacy instrument, the Crisis Resource Management Questionnaire [12]. This instrument has been shown to correlate with crisis resource management skills. It comprises four elements: situation awareness, team management, environment management and decision-making. Questionnaires were filled out and submitted anonymously. Pre and post training data were compared using a paired *t*-test.

## Results

All teams were monitored as to their success in managing each individual critical care scenario, as well as the 2–3.5 hour long maintenance of the casualty.

Trainees were scored using the evaluation forms presented in the Additional file 2. The CTD and observation sections were awarded 50 points each, divided evenly among the items. The average score in our training scenarios was 79.39%. Of the twelve simulated casualties, two expired in failed exercises. These two teams were debriefed in detail as to the reasons for expiration and how it may have been avoided. Successful management of the scenarios (meaning that the simulated patient survived the exercise) correlated with a score of 72% or above.

**Figure 1:**
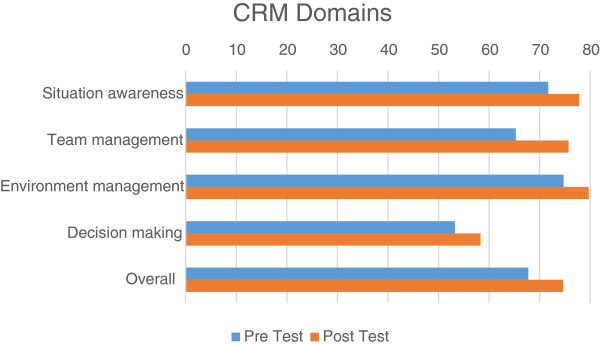
**CRM pre- and post-training scores.**

All twenty two trainees completed the CRM self-report questionnaire. The average Crisis Resource Management (CRM) self-efficacy score of the team leaders post exercise was 74.64% (2.27% standard deviation). Overall self-sufficiency scores improved by 10% following training (statistically significant improvement, p < 0.0001). Figure 1 displays pre- and post-training scores in the four CRM domains: situation awareness, team management, environment management and decision-making. A paired *t*-test shows a statistically significant improvement in all domains separately, and for the entire questionnaire (Table 2). Due to the small number of trainees we did not perform separate analyses for physicians and EMTs.

**Table 2:** **Average CRM scores**

	Pre-training (% score)	Post-training (% score)	P value
Situation awareness	71.68	77.85	0.0002
Team management	65.26	75.73	0.0003
Environment management	74.71	79.71	0.002
Decision making	53.23	58.29	<0.0001
Overall	67.74	74.64	<0.0001

## Discussion

The “Extended Evacuation Environment” is an emerging concept in military medical doctrine and so far relatively undescribed in the naval milieu. In contrast with prior doctrine whereby the trauma team would be deployed closer to the casualty or all efforts were made to shorten evacuation times, it is anticipated that immature, austere, outdistanced or depleted combat environments may pit a primary caregiver against complex and prolonged casualty management. It is imperative that relevant training modalities are developed for this emerging entity. We feel that we have taken some steps toward the realization of this goal. A pertinent question concurrent with “How to train” is certainly “What do we need to train?” Currently, the authors would humbly suggest that the realm of military extended prehospital care is underdeveloped and limited to catchy mnemonics (e.g. HITMAN) [3], basic ATLS/TCCC skills and existing equipment. The challenge for the next few years may be to develop a coherent set of guidelines and instruments for the primary caregiver experiencing extended evacuation scenarios.

Crisis resource management, an aviation concept often borrowed into emergency medicine, may be pertinent to the case of naval casualties. However, as medical CRM is far removed from the cockpit and requires some revision, care of naval casualties is even further removed. We therefore propose our own adaptation – NCM, or Naval Casualty Management. This is comprised of A. the ATLS/TCCC skills subset; B. The use of Crisis Resource Management tools for team leaders; C. Making contingencies for extended evacuation; and D. Damage control resuscitation. We have also began to establish a fifth foundation - the introduction of checklists in trauma.

The training of naval (or military) trauma teams poses ethical and practical challenges. Ethical considerations include those of best standards (i.e. of patient care and education), error management and patient safety, patient autonomy and the need to use live animals for training [13].

Practical considerations include the rarity of trauma casualties at sea during peace times (compared to a hospital setting for physicians in training) and the limited amount of time available for hospital-based training for enlisted medical personnel.

The training and evaluation modality presented differs from those formerly (and sometimes currently) used in common military medical simulators, i.e. simulation facilities in medical educational institutions [14, 15] or virtual reality [16, 17], where short scenarios are the standard. Our trainees were given extended care scenarios where a continuum of care was practiced, requiring a constant process of re-evaluation, diagnosis and treatment. Training was performed onboard a military naval vessel, in maximally realistic conditions, thus rehearsing a scenario of care in the genuine theater of operation. Medical immersion training is emerging as a teaching standard. Its use in medical simulations for training emergency care teams is expanding to civilian settings [18], military, including naval, settings [19], and the various domains of aerospace medicine [20–22].

A special and extreme case of austere environments may be found in spaceflight. As the traditional mentor/apprentice model of medical teaching is largely irrelevant for spacecraft crew medical officers, simulation and remote teaching modalities are gaining acceptance as possible and likely tools for skills training and maintenance. This may entail an effort to immerse the trainees in environment analogous to space, as can be seen for example in the experience described by Musson and Doyle in the Canadian Arctic Eureka weather station [20] or NASA’s use of medical simulators in microgravity, as described by Doerr et al. [22].

The various studies and experiences cited above all share characteristics similar to ours such as the austerity of the target environment or the cramped, noisy quarters involved (e.g. onboard a spaceship, helicopter or corvette). All made a point of proving the feasibility of deploying a simulator on their various platforms, and are in agreement that moving the teaching experience to the target environment is worthwhile. However, additional points present themselves from our experience for further consideration. For example, removal of the simulators from the training facility to the theater of operations has an added benefit. Simulation of battle injuries and prolonged treatment ratifies the adequacy and sufficiency of the medical instruments and supplies onboard that would otherwise only be tested in the case of actual casualties. A case in point: the first SUCCeSS exercise identified key medical supplies where modifications were necessary (e.g. length of IV extension tubes, number of cricothyroidotomy kits onboard, etc.). These were corrected before additional exercises and deployment, subsequently re-evaluated and found to be satisfactory.

A full script for a 3–4 hours scenario is impossible to write in advance and the patient’s situation may change in unforeseen ways following trainees’ management. It is therefore imperative that the instructor conducting the exercise be an experienced critical care physician who can logically change the condition of the simulated patient according to the actions of the trainees in a realistic manner (e.g. lowering blood pressure following an overzealous dose of certain anesthetics or inducing bradycardia if hypoxemia is not promptly dealt with). The presence of senior critical care specialist preceptors improved the level of teaching as they drew on rich medical and teaching experience and added credibility to the exercise.

In all our exercises, the CRM self-efficacy instrument displayed relatively high scores in team and environment management, with lower scores for situation awareness and decision-making. The latter may point to the physicians’ lower confidence in tasks related more directly to resuscitative care, emphasizing the need for such training sessions.

## Conclusion

Caregiver self-sufficiency and their self- perceived competence in treating casualties at sea was improved via high fidelity simulation in theatre using realistic naval casualty care situations. It is feasible to use medical simulation mannequins at sea, despite the logistic difficulties involved in their deployment. Ingenuity and enthusiasm during initial implementation may be required in order to overcome these. In light of the current technology and literature, we feel that striving for maximally realistic conditions in simulation at sea and elsewhere, should be the rule, not the exception.

**Figure 2:**
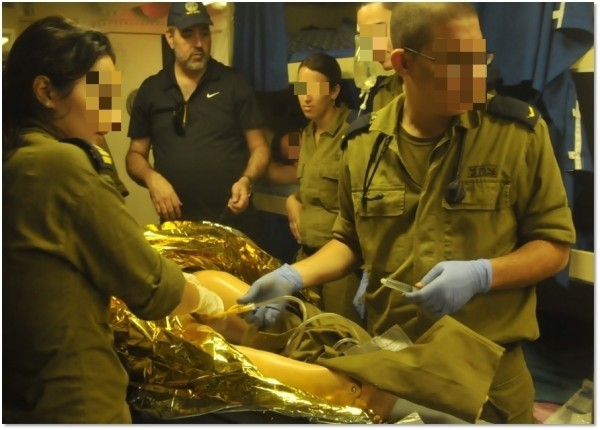
**Author DH (background, wearing navy cap) training a physician and EMT on board a corvette.** In the figure, a Foley catheter is being applied.

**Figure 3:**
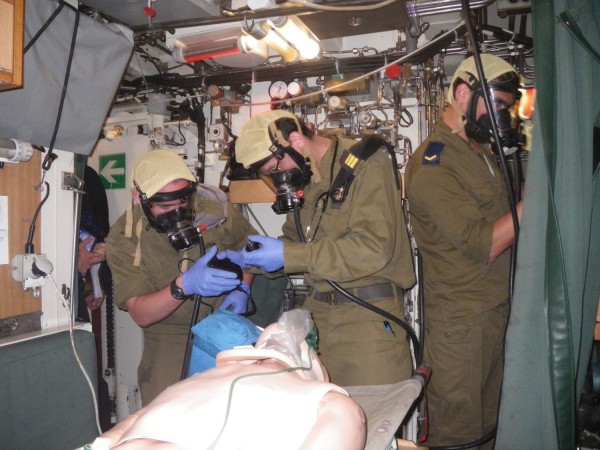
**A submarine surgeon and his team of medics is resuscitating a simulated patient after smoke inhalation.** The team is wearing standard breathing apparatus for flooding or smoke.

Limitations:

1. The simulation model is a plastic-silicone mannequin, without the tissue qualities of animal/cadaver models (e.g. in the performance of initial resuscitative procedures such as cricothyroidotomy) [23]. This may induce false confidence in the military physician’s actual skill level.
2. In the model used, certain clinical features were lacking, such as capillary refill, palpable body temperature, temperature differentials between limbs, perspiration and pupillary dynamics and bleeding, thus impeding the realism required from such drills. More advanced mannequins include some of these features.3. Artillery and gunfire were routinely heard during the corvette exercises, but we did not attempt to further simulate the emotional stress and physical conditions of naval battle. We feel that performing these exercises underway provides realistic naval conditions without causing excessive distraction. Figures 2 and 3 provide examples of onboard deployment on a corvette and submarine, respectively.
3. Additional training modalities and considerations that are regularly practiced elsewhere in the training of our personnel, but not concurrently within the SUCCeSS program:
  - Single model – our current program utilizes only a mannequin simulation model, without the use of live tissue or actors employing moulage.
  - The lack of a mass casualty event may be deemed as a pitfall as we focused on a single patient, in a single location, per training team. Our exercises began as multiple casualty for the sake of triage, and were later reduced to a single casualty.
  - No evacuation – the current program focuses only on the therapeutic aspect of Extended Prehospital Intensive Casualty Care, with no evacuation phases.
  - Teleconsultation – while this is well established for medical caregivers in the Israeli Navy, its use and training are beyond the scope of this paper.

### Information security

This text has been certified by the Information Security bureaus of the Israeli Navy and Techno-Logistic Division of the Israeli Defense Force as Unclassified.

## Appendix 1 Resuscitation devices and specifications

Resuscitation instruments included Uni-Vent 731 (IMPACT Instrumentation Inc. West Caldwell NJ, USA) and Versamed iVent201 (Versamed, GE Healthcare, Pearl River NY, USA) ventilators, Aitecs SEP-10S Plus syringe infusion pumps (Viltechmeda, Vilnius, Lithuania), and Nonin Model 9847 pulse-oxymeters/CO_2_ detectors (Nonin Medical Inc., Plymouth MN, USA). Blood pressure was measured using a manual sphygmomanometer; temperature was measured using standard digital oral thermometers capable of detecting hypothermia.

CO_2_ exhalation was simulated using pressurized tanks at a pressure of 120 bar (maximal outflow pressure regulated to 2 bar), connected to the mannequins through the SimMan control module. Medical oxygen was supplied using naval/aviation standard oxygen tanks. Other medications and instruments included the trauma standard supplied to missile ships and submarines, including medications for sedation, antibiotics, ACLS etc.

## Authors’ information

DH is a senior anesthesiologist and intensivist, and is a volunteer reservist in the IDF engaged in training and doctrine of naval medicine, forward resuscitation companies and ATLS. AW was formerly chief of the Trauma Instruction Section of the IDFMC Academy. He currently specializes in medical informatics and information technology. IN is a Lieutenant Commander in the Israeli Navy, and former Chief Surgeon of the Haifa Naval Base. He has also trained as an OB/GYN.

## Electronic supplementary material

Additional file 1: **Casualty no. 1-electrocution and head injury.** (DOCX )

Additional file 2: **Navy primary Caregiver CRM questionnaire.** (DOCX )
