# Supplementary material for: Naval casualty management training using human patient simulators
Source: Disaster Mil Med. 2015 Apr 6;1:9. doi: 10.1186/2054-314X-1-9 (PMC5330130; doi:10.1186/2054-314X-1-9)
Supplement: Supplementary file 1 — Additional file 1: Casualty no. 1-electrocution and head injury. (DOCX ) [file 40696_2014_9009_MOESM1_ESM.docx]

**Supplementary material I**

**Casualty no. 1 – electrocution and head injury**

Description: as the senior caregiver enters the room, the casualty is administered BLS by a combat medic who was on site during electrocution.

Moulage: entry and exit burns on both palms. Bleeding from head.

Casualty: Unconscious, no breathing or pulse. There is some bleeding from the scalp and palpation reveals deformation of the head.

| **Condition of the casualty** | **Required treatment** | **Complications** |
| --- | --- | --- |
| Unconscious, no pulse or breathing | Jaw thrust, bag ventilation, c-spine fixation.  Primary survey reveals no pulse -> continued BLS -> use of automatic defibrillator reveals VF -> electric defibrillation – sinus rhythm returns with only carotid pulse returning at this point | If not defibrillated -> death |
| Pulse returns, bradycardia, bradypnea, some moaning. Does not regain consciousness (GCS 4) | - Definitive airway - Ventilation with CO_2_ maintained at 35 mmHg due to increased ICP - Sedation: lidocaine, ketamine, midazolam. IF BP low additional midazolam drops BP - Mannitol administration | Vomiting, aspiration  Slowed respiration |
| Immobile for a long time.  Pale and with cold skin | - Heating of room, warm fluids, blanket - Position changes and padding | - Worsening of hypothermia - Pressure sores |
| Complementary monitoring:   - Sedation - Pupils - Urine output | - NG tube and foley catheter - Treatment of electrical burns - Warm fluid resuscitation - Medications: furosemide, bicarbonate, mannitol |  |
| Comments: consider raising head of bed by 30 degrees to reduce. |  |  |

Evaluation sheet – Casualty no. 1, electrocution and head injury

Name of trainee:

Additional observer:

Critical Treatment Decisions (CTDs): Did the trainee –

|  | yes | no |  |
| --- | --- | --- | --- |
| 1 | 🞏 | 🞏 | Correctly prioritize treatment |
| 2 | 🞏 | 🞏 | Display an organized approach to management |
| 3 | 🞏 | 🞏 | Perform evaluation, management and extended care well and in correct order |
| 4 | 🞏 | 🞏 | Identify and treat ventricular fibrillation |
| 5 | 🞏 | 🞏 | Manage definitive airway |
| 6 | 🞏 | 🞏 | Adjust ventilation and sedation for head injury |
| 7 | 🞏 | 🞏 | Identify hypothermia and take correct steps |
| 8 | 🞏 | 🞏 | Perform complementary treatment (antibiotics, hydration, raise head and torso) |
| 9 | 🞏 | 🞏 | Treat burns |

If the trainee ignores a CTD or manages it wrongly, consider deterioration and death of the casualty.

Observation of actions taken and their effect on the casualty

|  | not taken | deleterious | Indifferent | Beneficial |  |
| --- | --- | --- | --- | --- | --- |
| 1 | 🞏 | 🞏 | 🞏 | 🞏 | Use of capnometer/pulse-oxymeter |
| 2 | 🞏 | 🞏 | 🞏 | 🞏 | Mechanical ventilation |
| 3 | 🞏 | 🞏 | 🞏 | 🞏 | Warming the casualty |
| 4 | 🞏 | 🞏 | 🞏 | 🞏 | Secondary survey |
| 5 | 🞏 | 🞏 | 🞏 | 🞏 | Care and fixation of spine |
| 6 | 🞏 | 🞏 | 🞏 | 🞏 | Antibiotics administration |
| 7 | 🞏 | 🞏 | 🞏 | 🞏 | Sedation |
| 8 | 🞏 | 🞏 | 🞏 | 🞏 | Use of mannitol / hyperventilation |
| 9 | 🞏 | 🞏 | 🞏 | 🞏 | Preparation for evacuation |
| 10 | 🞏 | 🞏 | 🞏 | 🞏 | Use of NGT, foley, other tubes |

Comments:

Evaluation: 🞏 Succeeded 🞏Unsuccessful

**Casualty no. 2 – blast injury**

Description: The casualty is conscious, his voice is hoarse, is dyspneic. Burns on upper extremities, thorax and abdomen.

Moulage: Burns on upper body.

| **Condition of the casualty** | **Required treatment** | **Complications** |
| --- | --- | --- |
| Hoarseness, dyspnea | Immediate intubation.  Cricothyroidotomy if trismus | Bradycardia-> death |
| After 20 minutes: casualty exits sedation (blinks, trismus, motion).  SPO_2_% decrease  BP rises, airway pressure rises | Sedation medications using syringe pump (reduce BP if overdose).  Begin nursing care – NGT, foley, padding, bandages | If sedation administered by bolus, casualty inadequately sedated after 10 more minutes. If sedation not undertaken worsen vital signs. |
| After 1.5 hours: ventilation pressure rises, decreased ventilation left side,  SPO_2_% decrease (tension pneumothorax) | Needle thoracostomy  Thoracic drain | Deterioration -> death |
| Blood in chest drain – BP decreases  If hypothermic – cardiac arrhythmias (at 33°C) | Warm up the room  Warm hydration using Parkland formula  Check urine output | Ventricular arrhythmias. If hypovolemia not corrected->death |
| Immobile for a long time. | Prevention of pressure sores-padding, position changes every 2 hours | Pressure sores |
| Complementary treatment: antibiotics (consider anaphylaxis)  Treatment of burns  Tranexamic acid administration | Treatment of anaphylaxis | Deterioration if untreated, death |
|  | Comments:  Record treatment and responses  After intubation SPO_2_% does not exceed 90% (due to pulmonary contusion)  Energy depletion of battery operated devices | |

Evaluation sheet – Casualty no. 2 blast injury

Name of trainee:

Additional observer:

Critical Treatment Decisions (CTDs): Did the trainee –

|  | yes | no |  |
| --- | --- | --- | --- |
| 1 | 🞏 | 🞏 | Correctly prioritize treatment |
| 2 | 🞏 | 🞏 | Display an organized approach to management |
| 3 | 🞏 | 🞏 | Perform evaluation, management and extended care well and in correct order |
| 4 | 🞏 | 🞏 | Identify awakening from sedation, titrate medications |
| 5 | 🞏 | 🞏 | Identify breathing problems, pneumothorax |
| 6 | 🞏 | 🞏 | Perform needle thoracostomy and chest drain |
| 7 | 🞏 | 🞏 | Identify hypothermia and take correct steps |
| 8 | 🞏 | 🞏 | Perform complementary treatment (antibiotics, tranexamic acid) |
| 9 | 🞏 | 🞏 | Identify extended care complications (anaphylaxis, arrhythmias) and treat accordingly |

If the trainee ignores a CTD or manages it wrongly, consider deterioration and death of the casualty.

Observation of actions taken and their effect on the casualty

|  | not taken | deleterious | Indifferent | Beneficial |  |
| --- | --- | --- | --- | --- | --- |
| 1 | 🞏 | 🞏 | 🞏 | 🞏 | Use of capnometer/pulse-oxymeter |
| 2 | 🞏 | 🞏 | 🞏 | 🞏 | Mechanical ventilation |
| 3 | 🞏 | 🞏 | 🞏 | 🞏 | Warming the casualty |
| 4 | 🞏 | 🞏 | 🞏 | 🞏 | Secondary survey |
| 5 | 🞏 | 🞏 | 🞏 | 🞏 | Care and fixation of spine |
| 6 | 🞏 | 🞏 | 🞏 | 🞏 | Antibiotics administration |
| 7 | 🞏 | 🞏 | 🞏 | 🞏 | Sedation |
| 8 | 🞏 | 🞏 | 🞏 | 🞏 | Preparation for evacuation |
| 9 | 🞏 | 🞏 | 🞏 | 🞏 | Use of NGT, foley, other tubes |

Comments:

Evaluation: 🞏 Succeeded 🞏Unsuccessful
