# Supplementary material for: Naval casualty management training using human patient simulators
Source: Disaster Mil Med. 2015 Apr 6;1:9. doi: 10.1186/2054-314X-1-9 (PMC5330130; doi:10.1186/2054-314X-1-9)
Supplement: Supplementary file 2 — Additional file 2: Navy primary Caregiver CRM questionnaire. (DOCX ) [file 40696_2014_9009_MOESM2_ESM.docx]

**Navy primary Caregiver CRM questionnaire**

# Adapted from Plant JL, van Schaik SM, Sliwka DC, Boscardin CK, O'Sullivan PS. Validation of a self-efficacy instrument and its relationship to performance of crisis resource management skills. *Adv Health Sci Educ Theory Pract*. 2011;16(5):579-90.

*Please rate your level of agreement with the following statements by circling the response that most closely represents how you currently judge yourself in these areas*

**RATINGS: SD**=Strongly Disagree **D**=Disagree **N**=Neither Agree nor Disagree

**A**=Agree **SA**=Strongly Agree

| 1. ***In dealing with sick sailors, I am confident in my ability to*** |  |  |  |  |  |
| --- | --- | --- | --- | --- | --- |
| 1. recognize clinical deterioration | SD | D | N | A | SA |
| b. anticipate events | SD | D | N | A | SA |
| c. plan how to handle such events | SD | D | N | A | SA |
| ***2. When called to an emergency situation or***  ***code, I am confident in my ability to*** |  |  |  |  |  |
| a. gather information about the situation effectively | SD | D | N | A | SA |
| b. access additional resources (other health care  professionals) for additional help. | SD | D | N | A | SA |
| c. take charge as the team leader | SD | D | N | A | SA |
| ***As team leader in an emergency situation***  ***or code, I am confident in my ability to*** |  |  |  |  |  |
| a. follow ATLS algorithms | SD | D | N | A | SA |
| b. make decisions | SD | D | N | A | SA |
| c. see the big picture | SD | D | N | A | SA |
| d. consider a variety of explanations for the symptoms | SD | D | N | A | SA |
| e. identify a number of different possible interventions | SD | D | N | A | SA |
| f. decide on the most appropriate interventions | SD | D | N | A | SA |
| g. prioritize the necessary interventions | SD | D | N | A | SA |
| h. re-evaluate the situation and change plans as needed | SD | D | N | A | SA |
| i. delegate tasks appropriately | SD | D | N | A | SA |
| j. coordinate all team members | SD | D | N | A | SA |
| k. identify and utilize the skills of the team members | SD | D | N | A | SA |
| l. instruct and correct team members regarding their performance | SD | D | N | A | SA |
| m. elicit suggestions from other team members | SD | D | N | A | SA |
| n. communicate my plan clearly to the team | SD | D | N | A | SA |
| o. ensure that my requested interventions have taken place | SD | D | N | A | SA |
| p. provide reassurance and encouragement to the rest of the team | SD | D | N | A | SA |
| q. stay calm yourself | SD | D | N | A | SA |
| r. create and maintain a calm atmosphere among the team | SD | D | N | A | SA |
